# Synthesis, Spectral, and *In Vitro* Antibacterial Studies of Organosilicon(IV) Complexes with Schiff Bases Derived from Amino Acids

**DOI:** 10.1155/2013/425832

**Published:** 2013-07-28

**Authors:** Har Lal Singh, Jangbhadur Singh, A. Mukherjee

**Affiliations:** Department of Chemistry, Faculty of Engineering and Technology, Mody Institute of Technology and Science, Lakshmangarh, Sikar, Rajasthan 332311, India

## Abstract

The present work stems from our interest in the synthesis, characterization, and antibacterial evaluation of organosilicon(IV) complexes of a class of amino-acid-based Schiff base which have been prepared by the interaction of ethoxytrimethylsilane with the Schiff bases (N OH) in 1 : 1 molar ratio. These complexes have been characterized by elemental analysis, molar conductance, and spectroscopic studies including electronic IR and NMR (^1^H, ^13^C, and ^29^Si) spectroscopy. The analytical and spectral data suggest trigonal bipyramidal geometry around the silicon atom in the resulting complexes. The ligands and their organosilicon complexes have also been evaluated for *in vitro* antimicrobial activity against bacteria (*Bacillus cereus*, *Nocardia* spp., *E. aerogenes*, *Escherichia coli*, *Klebsiella* spp., and *Staphylococcus* spp.). The complexes were found to be more potent as compared to the ligands.

## 1. Introduction

In the last decade, coordination and organometallic compounds of biologically active ligands [1–3] have received much attention. However, it is notable that the biological activity of Schiff bases was significantly enhanced on chelation. It has been reported that chelation is the cause and cure of many diseases including cancer. Schiff base complexes [4–7] have found antibacterial, antifungal, anticancer, tuberculostatic, and herbicidal activities [8–12]. The current research dealing with metal complexes of heteronuclear Schiff bases has expanded enormously and includes diversified subjects comprising their various aspects in biocoordination and bioinorganic chemistry. It is known that the presence of metal ions bonded to biologically active compounds may enhance their activity [13–16]. Heteronuclear Schiff base complexes have found applications as magnetic materials, catalysts and in the field of bioengineering [17, 18]. Organosilicon compounds of nitrogen and sulphur containing ligands are well known for their anticarcinogenic, antibacterial, tuberculostatic, antifungal, insecticidal, and acaricidal activities [19–22]. The interest in organosilicon(IV) compounds [23–25] is due to their versatile applicability in the pharmaceutical industries. Generally, organosilicon compounds seem to owe their antitumour properties to the immune-defensive system of the organism. The medical applications and effectiveness of the silatranes in the treatment of wounds and tumours are thought to be related to the role of silicon in the growth of epithelial and connective tissues and hair, where their function is to impart strength, elasticity, and impermeability to water [26]. 

In view of this, the synthesis of organosilicon(IV) complexes of Schiff bases derived from the condensation of chloroisatin and isatin with different amino acids derivatives is reported herein. The characterization of the complexes was realised by elemental analysis and spectroscopic (UV, IR, ^1^H, ^13^C, and ^29^Si NMR) studies. Their antibacterial activities were screened against various bacteria.

## 2. Experiment

Adequate care was taken to keep the organosilicon(IV) complexes, chemicals, and glass apparatus free from moisture; clean and well-dried glass apparatus fitted with quickfit interchangeable standard ground joints was used throughout the experimental work. All the chemicals and solvents used were dried and purified by standard methods. The ligands were prepared by the condensation of isatins with amino acids as described earlier [27, 28].

### 2.1. Physical Measurements and Analytical Methods

Silicon was determined gravimetrically as SiO_2_. Nitrogen and sulphur were estimated by Kjeldahl's and Mesenger's methods, respectively. Molecular weights were determined by the Rast camphor method (freezing point depression method) using resublimed camphor (MP 178°C). The conductance measurements were carried out in dry dimethylformamide (DMF) at room temperature using a systronics conductivity bridge (model 305) in conjunction with a cell having a cell constant of 1.0. The electronic spectra were recorded on a Thermo UV1 visible spectrophotometer in the range 200–800 nm, using dry methanol as the solvent. Infrared spectra were recorded on a Perkin Elmer, FT-IR SP-2 spectrophotometer in KBr pellets. Multinuclear magnetic resonance spectra were recorded on BRUKER AVANCE II FTNMR 400 MHz spectrometer. ^1^H NMR spectra were recorded in deuterated dimethylsulphoxide (DMSO-d_6_) at 400 MHz using tetramethylsilane (TMS) as an internal standard. ^13^C and ^29^Si NMR spectra were recorded in dry dimethylsulphoxide using TMS as the internal standard.

### 2.2. Synthesis of the Organosilicon(IV) Complexes

The complexes were prepared under anhydrous conditions by the slow addition of a dry, hot methanol solution of the ethoxytrimethylsilane (0.47 g; 3.385 mmole) in a 1 : 1 molar ratio to a solution of the Schiff bases (0.691–1.127 g; 3.385 mmole) in dry methanol (60 mL). The mixture was refluxed with constant stirring, giving a clear solution in half an hour; refluxing was then continued for 10–12 hr. Excess solvent was removed under reduced pressure, and the compound was finally dried in vacuum at a bath temperature of 40 ± 5°C on rotary evaporator after being repeatedly washed with a mixture of methanol and *n*-hexane (1 : 1 v/v). The crystalline solids were separated out and purified by recrystallization from the same solvent. The purity of the compounds was checked by TLC using silica gel-G as adsorbent. Their physical properties and analytical data are recorded in Table 1.

### 2.3. Antibacterial Assay

Synthesized compounds were screened for their antibacterial activity against *Bacillus cereus, Nocardia* spp., *E. aerogenes, Escherichia coli, Klebsiella* spp.,* and Staphylococcus* spp. at the concentrations of 100 *μ*g/mL by the agar well-diffusion method [29]. An aliquot (5 mL) of nutrient broth was inoculated with the test organisms and incubated at 37°C for 24 h. Sterile nutrient agar plates were also prepared, and holes of 6 mm diameter were cut using a sterile cork borer ensuring proper distribution. The test organisms after 24 h of incubation were spread onto separate agar plates. The compounds were dissolved in DMSO and were poured into appropriately labeled holes using a pipette in aseptic conditions. DMSO served as control with Streptomycin (100 *μ*g/mL) used as a standard antibiotic. whole determination was made in triplicate for each of the compounds. An average of three independent readings for each compound was recorded. The zone of inhibition was calculated in millimeters carefully.

## 3. Results and Discussion

**Scheme 1:**
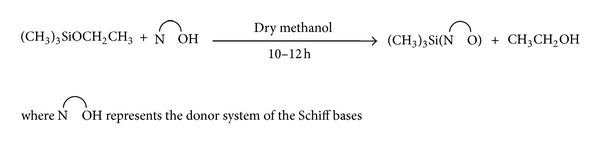
Representative equation illustrating the formation of Me_3_Si(L) complexes.

The 1 : 1 molar reactions of Me_3_Si (OC_2_H_5_) with Schiff base of amino acids have led to the formation of Me_3_Si(L) type of complexes. The reactions have been carried out in dry methanolic medium. These reactions can be represented by the general equations in Scheme 1 showing the formation of the complexes.

All the newly synthesized organosilicon(IV) complexes were coloured solids soluble in DMSO, DMF, and methanol. The compounds were dissolved in DMF and molar conductance 10^−3 ^M of solution at 45°C was measured. The molar conductance valves of the complexes fall in the range 08–16 ohm^−1 ^cm^2 ^mol^−1^, indicating that these compounds are nonelectrolytic nature. The analytical data were in the good agreement with the proposed stoichiometry of the complexes (Table 1). 

### 3.1. Electronic Spectra

**Figure 1:**
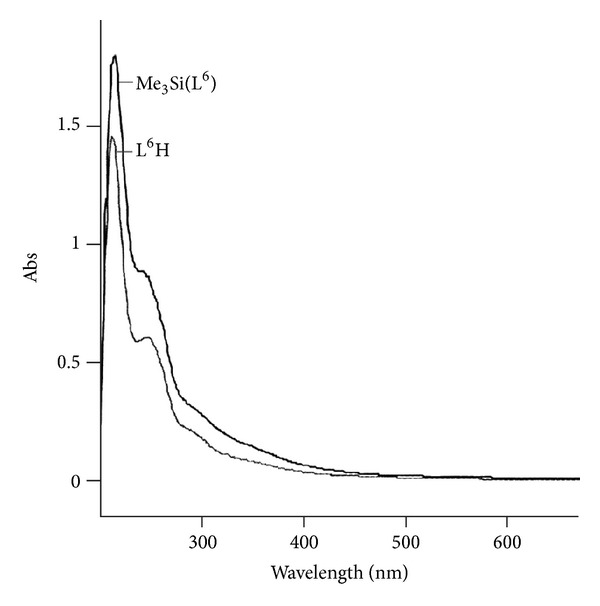
Electronic Spectra of Ligand (L^6^H) and their Silicon Complex.

The electronic spectra of the Schiff base and its 1 : 1 organosilicon(IV) complexes have been recorded in methanol (Figure 1). Complexes exhibit two bands in the regions 205–220 and 250–260 nm, which may be due to the *π*–π* transition of benzenoid/*π*–π* transition of COO, chromophore, respectively. The spectra of the ligand show a weak broad absorption band at ~340 nm which can be assigned to the *n*-π* transitions of the azomethine group. This band shows a blue shift in the silicon complexes appearing at ~332 nm, due to the polarisation within the >C=N− chromophore caused due to formation of covalent silicon-nitrogen bond. The bands at ~260 and ~282 nm are due to *π*–π* transitions, within the benzene ring and (>C=N−) band of the azomethine group, respectively. The K band *π*–π* showed a red shift due to the overlap of the central silicon d-orbital with the p-orbital of the donor atom which causes an increase in conjugation, and the B-bands undergo a hypsochromic shift in the complexes.

### 3.2. IR Spectra

The characteristic infrared absorption frequencies (in cm^−1^) and their assignments for the ligands and their organosilicon(IV) complexes are given in Table 2. The assignments of characteristic IR frequencies for the resulting complexes may be discussed as follows. The IR spectra of these derivatives do not show any band in the region 3110–2740 cm^−1^ which could be assigned to *ν*(COOH). This clearly indicates the deprotonation of the ligand as a result of complexation with the silicon atom [28]. It is further confirmed by the appearance of sharp band at 520–535 cm^−1^ in the spectra of all the complexes assignable to the *ν*(Si–O) stretching vibrations [30]. In the spectra of the complexes two sharp bands are observed at 1604 and 1320 cm^−1^ and are assigned to the *ν*_asym_(COO) and *ν*_sym_(COO), respectively. Furthermore, the separation between asymmetric and symmetric vibrations is about 271 ± 5 cm^−1^, indicating the covalent nature of the silicon-oxygen bond. Ionic bonding and also bridging/chelation can therefore be excluded, and it must be assumed that the carboxylic group binds silicon unidentally. Moreover, Δ*ν* values of complexes below 200 cm^−1^ would be expected for bridging or chelating carboxylates but greater than 200 cm^−1^ for the monodentate bonding carboxylate anions [31, 32]. The C=O band of the indole group appears in the range of 1720–1735 cm^−1^ in the ligands. However, a strong band at ~1730 cm^−1^ due to the vibration of C=O group remains unchanged in the spectra of complexes showing thereby the noninvolvement of this group in coordination, thus confirms that the C=O from indole is not involved in the complexation. 

The sharp and strong band at 1628 ± 7 cm^−1^ due to *ν*(>C=N−) frequency of the free azomethine group in the ligand shifts to the lower frequency (10–15 cm^−1^) in the spectra of the silicon complexes, indicating coordination through the azomethine nitrogen to the silicon atom. The shift can be explained by a reduction of the carbon-nitrogen double bond character in the azomethine group [33, 34]. Formation of a silicon nitrogen bond was further confirmed by the presence of a new band at 570–550 cm^−1^ of *ν*(Si*←*N) [35]. A characteristic band at 3250 cm^−1^, due to *ν*(N–H) of indole, was observed in the spectra of the ligand and their silicon complexes. Several new bands of strong to medium intensity in the spectra of the complexes at 1272 ± 5 and 760 ± 5 cm^−1^ may be due to the asymmetric deformation mode of Si–CH_3_ and stretching vibrations of Si–C, respectively [36, 37].

### 3.3. ^1^H NMR Spectra

The ^1^H NMR spectral data of the ligands and their silicon complexes have been recorded in DMSO-d_6_. The chemical shift values relative to the TMS peak are listed in Table 3. The ^1^H NMR spectral data of the ligands show single resonance at *δ* 11.25–12.55 ppm, which is absent in the spectra of the silicon complexes, indicating the replacement of the carboxylic protons by the Si(IV) moiety. The ligand shows a complex pattern in the region *δ* 8.10–6.92 ppm for the aromatic protons, and this is observed in the region *δ* 7.95–7.10 ppm in the spectra of the organosilicon(IV) complexes. This shifting also supports the coordination through the nitrogen atom. The appearance of signals due to NH protons at the same positions in the ligand and its complexes shows the noninvolvement of this group in coordination. Schiff bases derived from glycine, alanine, valine, and methionine display four/three aromatic protons, as expected. In the spectrum of phenylalanine, the integral of the aromatic region corresponds to nine protons; five protons on the phenyl ring are recognizable at 7.4 ppm. Methylene (for glycine, alanine, valine, and methionine) protons on the *α*-carbon of the carboxylic acid moieties appear at *δ* 3.96–4.30 ppm. This signal is a singlet for (**1**), a doublet for (**3**) and (**6**), a triplet for (**7**), and (**8**) and a quartet for (**2**) and (**5**) all of which arise from the nonequivalent methylene protons in structures (**1**–**8**). In general, the complexes obtained were found to exhibit no additional resonances and thus reflect the purity of the complexes. The integration of peaks concurs with the number of protons postulated from the structures proposed for the complexes. The additional signal in the region *δ* (1.32−1.20 ppm) in Me_3_Si(L) complexes is due to Me_3_Si group. 

### 3.4. ^13^C NMR Spectra

The ^13^C-NMR spectral data along with assignment of characteristic signals of ligands and its organosilicon(IV) complexes are presented in Table 4. The signals due to the carbon atoms attached to the carboxylate and the azomethine groups in ligands appear at *δ* 176.1–178.5 ppm and *δ* 155.8–163.6 ppm, respectively. However, in the spectra of the corresponding silicon complexes, these appear at *δ* 180.7–186.1 ppm (carboxylate group) and at *δ* 150.2–152.7 ppm (azomethine group), respectively. The considerable shifts in the positions of these signals clearly indicate the involvement of these functional groups in bond formation with the silicon atom. The carbon of methyl groups (Si–CH_3_) is observed at position comparable to other similar compounds. The occurrence of resonances in the range of *δ* 118.3–150.7 ppm in the ^13^C-NMR spectra of the complexes and ligand was defined as aromatic carbon signals.

Although it is also possible that the shifting of the azomethine carbon signal and carboxylate carbon signal is because of a change in hybridization of the nitrogen and oxygen attached to the group, in the light of IR, UV, and ^1^H NMR spectral studies it seems more plausible that the shifting in these carbons is due to the involvement of carboxylate oxygen and azomethine nitrogen in bonding.

### 3.5. ^29^Si NMR Spectra

**Figure 2:**
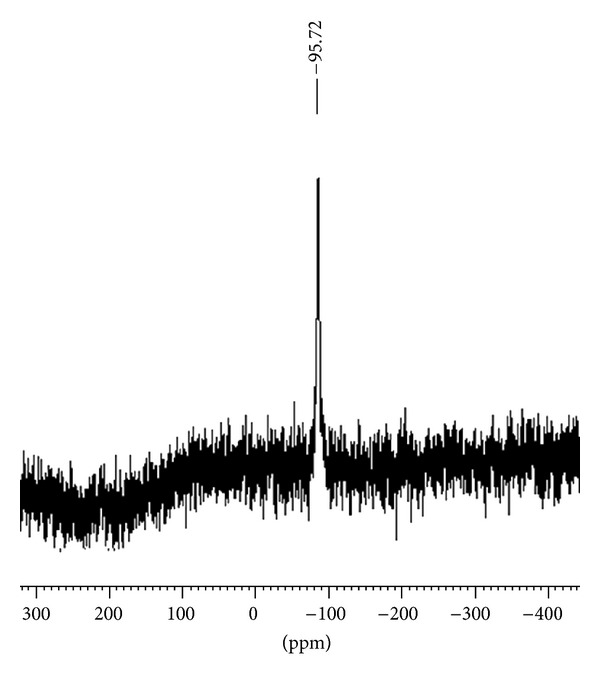
^29^Si NMR spectrum of Me_3_Si(L^2^).

**Figure 3:**
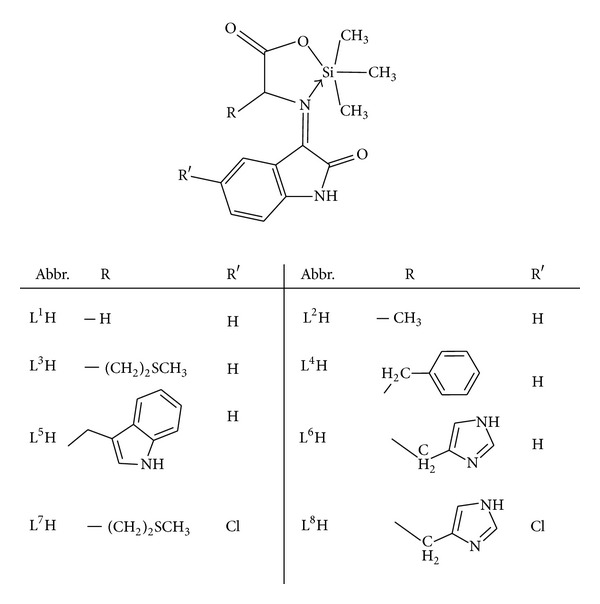
Structure of Organosilicon(IV) Complexes.

In order to confirm the geometry of the complexes, ^29^Si NMR spectra of the complexes were recorded (Figure 2). The value of *δ* ^29^Si in the spectra reflects the coordination number of the nucleus in the corresponding silicon complexes [38, 39]. In general, ^29^Si chemical shift moves to lower frequency with increasing coordination number of the nuclei. The spectra show in each case only one sharp singlet indicating the formation of a single species. In the ^29^Si NMR spectra of the silicon complexes, a sharp signal appears in the range of −96.8 to −90.9 ppm with respect to TMS indicating a penta-coordinated environment around the silicon atom with the nitrogen atom occupying equatorial position and the most electronegative atom occupying axial position. On the basis of the spectroscopic studies, the penta-coordinated structure of the complexes shown in Figure 3 has been proposed.

### 3.6. Antimicrobial Activities

*In vitro* antibactericidal activity of the ligands (HL^1–4^), silicon complexes, and standard drugs was screened separately for their antibacterial activity against Gram-positive and Gram-negative bacteria (*Bacillus cereus, Nocardia *spp.,* E. aerogenes, Escherichia coli, Klebsiella *spp., *and Staphylococcus *spp.). Streptomycin was used as a reference compound for antibacterial activities. These bacterial strains are used because they are known as common pathogens of human beings. The antimicrobial studies suggested that the Schiff bases are biologically active and their silicon complexes showed significantly enhanced antibacterial activity against microbial strains in comparison to the free ligands. Tested compounds showed zone of inhibition ranging 5.6 mm–12.9 mm against the Gram-positive bacteria and between 2.8 mm–10.8 mm against Gram-negative bacteria. The ligands (HL^1–4^) show zone of inhibition ranging 5.6 mm–9.9 mm against Gram-positive bacteria and 2.8 mm–7.4 mm against Gram-negative bacteria. It has been observed that the silicon complexes showed increased zone of inhibition against the bacterial strains (Table 5) as compared to ligands. On the basis of zone of inhibition produced against the test bacterium, compound 3 was found to be most effective against *Bacillus cereus, Nocardia *spp.,* Staphylococcus *spp., *E. coli, Klebsiella *spp., and* E. aerogenes* with zone of inhibition of 12.6 mm, 12.7 mm, 12.9 mm, 9.5 mm, 9.9 mm, and 8.7 mm, respectively (Table 5). This also showed that the antibacterial activity of ligands is greatly enhanced when it is coordinated to silicon ions.

Although it is difficult to make out an exact structure-activity relationship between the antimicrobial activity and the structure of these complexes, it can possibly be concluded that the biological activity of the ligands exhibited a marked enhancement on coordination with the silicon ions against all the test bacterial strains which shows that silicon chelates are more active than the ligands. This may be explained by Tweedy's chelation theory [40, 41] according to which chelation reduces the polarity of the central metal atom because of the partial sharing of its positive charge with the ligand, which favours permeation of the complexes through the lipid layer of cell membrane [42]. According to the Overtone's concept of cell permeability, the lipid membrane surrounding the cell favors the passage of only lipid-soluble materials; therefore, liposolubility is an important factor which controls the antimicrobial activity [43]. On chelation, polarity of the metal ion is reduced to a greater extent due the overlapping of the ligand orbital and partial sharing of the positive charge of the silicon ion with donor groups. Moreover, delocalization of the p-electrons over the whole chelate ring is increased and lipophilicity of the complexes is enhanced. The increased lipophilicity enhances the penetration of the complexes into the lipid membranes and blocks the silicon binding sites in the enzymes of microorganisms. In general, silicon complexes are more active than ligands as they may serve as principal cytotoxic species.

## 4. Conclusions

We report here the synthesis and the characterization of eight new complexes of silicon with Schiff bases derived from isatins and amino acids. The newly synthesized Schiff bases act as bidentate ligands coordinating to silicon ion through azomethine nitrogen and carboxylate oxygen atom. The synthesized compounds were characterized by elemental analysis, UV-visible, IR and NMR spectroscopy, as well as by conductance measurements. Thus, on the basis of the previously mentioned spectral features, as well as the analytical data, the penta-coordinated trigonal bipyramidal geometries shown in Figure 3 have been suggested for the organosilicon(IV) complexes. The Schiff bases and their silicon complexes were found to be highly active against some of the antibacterial species. The activity is significantly increased on coordination.

## Figures and Tables

**Table 1:** Analytical and physical data of the Me_3_Si(IV) complexes.

C. no	Products and colour	M.P. °C	Yield (%)	Elemental analysis, Found (Calcd.)					Mol. Wt. Found (Calcd.)
				% Si	% C	% H	% N	% S	
Me_3_SiL^1^	C_13_H_16_N_2_O_3_Si reddish	104	69.55	10.01 (10.16)	56.45 (56.50)	5.82 (5.84)	10.19 (10.14)	—	270.65 (276.36)
Me_3_SiL^2^	C_14_H_18_N_2_O_3_Si reddish	138	65.36	9.50 (9.67)	57.84 (57.90)	6.27 (6.25)	9.60 (9.65)	—	281.98 (290.39)
Me_3_SiL^3^	C_16_H_22_N_2_O_3_SSi reddish	116	69.80	8.15 (8.01)	54.75 (54.83)	6.30 (6.33)	7.92 (7.99)	9.01 (9.15)	356.12 (350.51)
Me_3_SiL^4^	C_20_H_22_N_2_O_3_Si reddish	82	79.59	7.56 (7.66)	65.63 (65.55)	6.02 (6.05)	7.65 (7.64)	—	357.83 (366.49)
Me_3_SiL^5^	C_22_H_23_N_3_O_3_Si brown	160	73.43	6.85 (6.93)	65.02 (65.16)	5.69 (5.72)	10.30 (10.36)	—	413.11 (405.52)
Me_3_SiL^6^	C_17_H_20_N_4_O_3_Si brown	132	90.30	7.93 (7.88)	57.19 (57.28)	5.60 (5.66)	15.75 (15.72)	—	350.94 (356.45)
Me_3_SiL^7^	C_16_H_21_ClN_2_O_3_SSi reddish	122	56.09	7.18 (7.30)	49.83 (49.92)	5.45 (5.50)	7.21 (7.28)	8.30 (8.33)	380.32 (384.95)
Me_3_SiL^8^	C_17_H_19_ClN_4_O_3_Si brown	130	76.65	7.06 (7.18)	52.33 (52.23)	4.88 (4.90)	14.23 (14.33)	—	383.56 (390.90)

**Table 2:** Important IR spectral data (cm^−1^) of Schiff bases and their corresponding organosilicon(IV) complexes.

Compounds	*ν* (OH)	*ν* (C=N–)	*ν* (C=O)	*ν* (COO)_asym_	*ν* (COO)_sym_	Δ*ν*	*ν* (Si*←*N)	*ν* (Si–O)
L^1^H	3,090–2750 br	1625 s	1,720 s	—	—	—	—	—
Me_3_SiL^1^	—	1610 m	1722 m	1588 vs	1322 m	266	570 m	425 m
L^2^H	3105–2790 br	1635 s	1730 s	—	—	—	—	—
Me_3_SiL^2^	—	1622 s	1729 s	1595 vs	1320 s	275	550 w	430 m
L^3^H	3090–2750 br	1625 s	1720 s	—	—	—	—	—
Me_3_SiL^3^	—	1614 s	1720 s	1604 s	1332 s	272	555 s	420 w
L^4^H	3090–2740 br	1620 s	1728 m					
Me_3_SiL^4^		1608 m	1726 m	1590 s	1318 m	272	550 m	428 m
L^5^H	3108–2795 br	1630 s	1725 s	—	—	—	—	—
Me_3_SiL^5^	—	1615 s	1724 s	1594 s	1324 s	270	568 m	429 s
L^6^H	3090–2740 br	1620 s	1728 s	—	—	—	—	—
Me_3_SiL^6^	—	1609 s	1730 s	1600 s	1324 m	276	558 m	427 w
L^7^H	3110–2750 br	1622 s	1735 s	—	—	—	—	—
Me_3_SiL^7^	—	1611 s	1732 s	1597 vs	1322 s	275	560 w	425 w
L^8^H	3100–2740 br	1625 s	1730 m	—	—	—	—	—
Me_3_SiL^8^	—	1610 m	1732 s	1592 s	1320 m	272	565 m	435 m

br: broad, vs: very sharp, v: sharp, m: medium, and w: weak.

**Table 3:** ^
1^H NMR spectral data^a^ of the ligands and their corresponding Me_3_Si(IV) complexes.

Compounds	Chemical Shift (*δ*, ppm)
L^1^H	11.28 (s, 1H, COOH), 4.30 (s, 2H, N–CH_2_–), 8.02 (s, 1H, NH), H-aromatic: 7.58 (d, *J* = 7.8, 1H), 7.28–7.48 (m, 2H,), and 7.80 (d, *J* = 7.7, 1H).
Me_3_SiL^1^	4.32 (s, 2H, N–CH_2_–), 8.03 (s, 1H, NH), H-aromatic: 7.72 (d, *J* = 7.6, 1H), 7.22–7.52 (m, 2H,), 7.98 (d, *J* = 7.3, 1H), and 1.32 (s, 9H, Si–CH_3_)
L^2^H	11.25 (s, 1H, COOH), 4.72 (q, 1H, CH), 8.34 (s, 1H, sec. amide), 1.31 (d, *J* = 6.8, 3H, CH_3_), H-aromatic: 7.61 (d, *J* = 7.6, 1H), 7.21–7.41 (m, 2H,), and 7.82 (d, *J* = 7.4, 1H).
Me_3_SiL^2^	4.70 (q, 1H, CH), 8.36 (s, 1H, sec. amide), 1.28 (d, 3H, CH_3_), H-aromatic: 7.66 (d, *J* = 7.7, 1H), 7.20–7.46 (m, 2H,), 7.98 (d, *J* = 7.6, 1H), and 1.25 (s, 9H, Si–CH_3_)
L^3^H	11.74 (s, 1H, COOH), 4.72 (d, *J* = 6.8, 1H, CH), 8.05 (s, 1H, sec. amide), 2.30 (m, 2H, CH_2_), 2.04 (d, *J* = 6.4, 3H, CH_3_), H-aromatic: 7.58 (d, *J* = 8.1, 1H), 7.18–7.38 (m, 2H,), and 7.72 (d, *J* = 7.6, 1H).
Me_3_SiL^3^	4.50 (d, 1H, CH), 8.01 (s, 1H, sec. amide), 2.22 (m, 2H, CH_2_), 2.06 (d, 3H, CH_3_), H-aromatic: 7.64 (d, *J* = 7.9, 1H), 7.16–7.46 (m, 2H,), 7.88 (d, *J* = 7.5, 1H), and 1.26 (s, 9H, Si–CH_3_)
L^4^H	11.50 (s, 1H, COOH), 4.25 (t, 1H, N–CH–CH_2_–), 3.08 (d, *J* = 6.8, 2H, –CH_2_–Ph), 8.01 (s, 1H, NH), ), H-aromatic: 7.62 (d, *J* = 8.1, 1H), 7.20–7.45 (m, 2H,), and 7.80 (d, *J* = 7.8, 1H).
Me_3_SiL^4^	4.26 (t, 1H, N–CH–CH_2_–), 3.12 (d, *J* = 6.9, 2H, –CH_2_–Ph), 8.08 (s, 1H, NH), H-aromatic: 7.70 (d, *J* = 7.9, 1H), 7.20–7.45 (m, 2H,), 7.98 (d, *J* = 7.8, 1H), and 1.30 (s, 9H, Si–CH_3_)
L^5^H	11.46 (s, 1H, COOH), 4.39 (d, *J* = 8.3, 1H, CH), 8.12 (s, 1H, sec. amide), 10.15 (s, 1H, indole), 3.02 (m, 2H, CH_2_), H-aromatic: 7.65 (d, *J* = 7.8, 1H), 7.15–7.30 (m, 2H,), and 7.84 (d, *J* = 7.6, 1H).
Me_3_SiL^5^	3.16 (d, 1H, CH), 8.07 (s, 1H, sec. amide), 10.18 (s, 1H, indole), 2.98 (m, 2H, CH_2_), H-aromatic: 7.64 (d, *J* = 7.7, 1H), 7.18–7.42 (m, 2H,), 8.01 (d, *J* = 7.6, 1H), and 1.25 (s, 9H, Si–CH_3_)
L^6^H	12.55 (s, 1H, COOH), 4.61 (d, *J* = 7.2, 1H, CH), 8.10 (s, 1H, sec. amide), 12.92 (s, 1H, imidazole), 3.15 (m, 2H, CH_2_), H-aromatic: 7.60 (d, *J* = 7.9, 1H), 7.08–7.38 (m, 2H,), and 7.80 (d, J = 7.7, 1H).
Me_3_SiL^6^	3.98 (d, 1H, CH), 8.03 (s, 1H, sec. amide), 12.79 (s, 1H, imidazole), 3.10 (m, 2H, CH_2_), 7.62 (d, *J* = 7.8, 1H), 7.10–7.44 (m, 2H), 7.94 (d, *J* = 7.7, 1H), and 1.28 (s, 9H, Si–CH_3_)
L^7^H	11.72 (s, 1H, COOH), 4.80 (d, *J* = 6.9, 1H, CH), 8.15 (s, 1H, sec. amide), 2.30 (m, 2H, CH_2_), 2.10 (d, *J* = 6.5, 3H, CH_3_), H-aromatic: 7.56 (d, *J* = 7.7, 1H), 6.95–7.36 (m, 2H,), and 7.76 (d, *J* = 7.8, 1H).
Me_3_SiL^7^	4.86 (d, *J* = 6.9, 1H, CH), 3.10 (m, 1H, CH), 8.10 (s, 1H, sec. amide), 2.25 (m, 2H, CH_2_), 2.02 (d, *J* = 6.3, 3H, CH_3_), H-aromatic: 7.66 (d, *J* = 7.7, 1H), 7.12–7.48 (m, 2H,), 7.95 (d, *J* = 7.9, 1H), and 1.20 (s, 9H, Si–CH_3_)
L^8^H	12.28 (s, 1H, COOH), 4.45 (t, 1H, –CH–), 3.28 (d, *J* = 6.5, 2H, CH_2_), H-aromatic: 7.60 (d, *J* = 7.8, 1H), 7.12–7.38 (m, 2H,), and 7.82 (d, *J* = 7.7, 1H).
Me_3_SiL^8^	4.40 (t, 1H, –CH–), 3.25 (d, *J* = 6.6, H, CH_2_), H-aromatic: 7.65 (d, *J* = 7.8, 1H), 7.14–7.50 (m, 2H,), 7.92 (d, J = 7.8, 1H), and 1.27 (s, 9H, Si–CH_3_)

^a^Chemical shift (*δ*) in ppm: multiplicity is given as s: singlet, d: doublet, t: triplet, q: quartet, and m: complex pattern.

**Table 4:** ^
13^C NMR spectral data of the ligands and their corresponding organosilicon(IV) complexes.

Compounds	Chemical shift in (*δ* ppm)				
	COOH	CH	C=N	Si–CH_3_	Aromatic carbons
L^1^H	176.1	52.4	163.6	—	148.5, 131.0, 129.8, 125.3, 122.9, 119.1
Me_3_SiL^1^	185.4	53.3	152.4	14.10	145.3, 131.2, 129.5, 125.1, 122.6, 120.2
L^2^H	172.6	63.5	155.8	—	159.9, 130.4, 129.5, 133.8, 110.9, 148.5, 121.3
Me_3_SiL^2^	180.7	64.2	150.2	13.67	155.4, 130.6, 129.2, 134.0, 111.5, 148.1, 121.0
L^3^H	178.2	60.4	162.9	—	157.3, 144.6, 136.1, 132.3, 128.4, 122.5, 116.8
Me_3_SiL^3^	186.1	60.1	152.6	13.90	155.9, 144.9, 135.9, 132.3, 129.1, 122.3, 117.2
L^4^H	176.8	67.8	163.4	—	149.2, 135.8, 133.0, 128.6, 127.5, 126.3, 124.6, 122.9, 120.1
Me_3_SiL^4^	184.8	66.6	151.3	13.90	146.7, 135.9, 133.2, 127.3, 127.0, 126.5, 124.8, 122.3, 120.4
L^5^H	177.4	72.6	162.4	—	160.2, 150.7, 138.1,131.5, 130.4, 128.2, 124.5, 124.1,123.8, 122.6, 121.2, 120.6, 117.4, 112.2, 109.4
Me_3_SiL^5^	184.7	72.3	152.7	13.98	158.1, 149.6, 138.0, 131.7, 130.1, 128.7, 124.8, 124.4, 123.4, 122.3, 121.6, 120.1, 118.8, 112.9, 110.3

**Table 5:** Antibacterial activity of ligands and their organosilicon(IV) complexes.

Compounds	Inhibition zone (mm) and activity index (AI)^a^					
	*Bacillus cereus *	*Nocardia *spp.	*Staphylococcus *spp.	*E. coli *	*Klebsiella *spp.	*E. aerogenes *
	100 *μ*g/mL	100 *μ*g/mL	100 *μ*g/mL	100 μg/mL	100 *μ*g/mL	100 *μ*g/mL
L^1^H	5.6 (0.394)	5.9 (0.450)	6.7 (0.453)	Inact.	Inact.	Inact.
Me_3_SiL^1^	7.8 (0.549)	8.4 (0.641)	8.2 (0.554)	3.6 (0.283)	2.8 (0.250)	3.5 (0.280)
L^2^H	6.6 (0.465)	7.2 (0.550)	7.9 (0.534)	Inact.	Inact.	Inact.
Me_3_SiL^2^	10.5 (0.739)	11.9 (0.908)	9.8 (0.662)	3.9 (0.307)	3.2 (0.286)	3.5 (0.280)
L^3^H	9.6 (0.394)	9.9 (0.556)	9.4 (0.635)	6.7 (0.528)	5.4 (0.482)	6.2 (0.496)
Me_3_SiL^3^	12.6 (0.887)	12.7 (0.969)	12.9 (0.871)	9.5 (0.748)	9.9 (0.884)	8.7 (0.696)
L^4^H	8.5 (0.599)	8.1 (0.618)	8.7 (0.587)	6.2 (0.488)	7.4 (0.661)	7.1 (0.568)
Me_3_SiL^4^	10.6 (0.745)	12.4 (0.947)	12.1 (0.818)	9.8 (0.772)	9.5 (0.848)	10.8 (0.864)
Streptomycin	14.2	13.1	14.8	12.7	11.2	12.5

^a^(AI): inhibition zone of test compounds/inhibition zone of standard.
